# +50 Years of Terrestrial Hydroclimatic Variability in Africa’s Transboundary Waters

**DOI:** 10.1038/s41598-019-48813-x

**Published:** 2019-08-23

**Authors:** Emad Hasan, Aondover Tarhule, Joseph T. Zume, Pierre-Emmanuel Kirstetter

**Affiliations:** 1 Department of Geography, State University of New York, SUNY at Binghamton, Vestal, NY USA; 2 Geography and Earth Science Department, Shippensburg University, Shippensburg, PA USA; 3 Hydrometrology and Remote Sensing (HyDROS) laboratory, University of Oklahoma, Norman, OK USA; 4 School of Meteorology, University of Oklahoma, Norman, OK USA; 5 School of Civil Engineering and Environmental Science, University of Oklahoma, Norman, OK USA; 6 NOAA/National Severe Storms Laboratory, University of Oklahoma, Norman, OK USA; 7 Geology Department, Faculty of Science, Damietta University, New Damietta, Egypt

**Keywords:** Hydrology, Hydrology

## Abstract

GRACE Terrestrial Water Storage (TWS) provides unique and unprecedented perspectives about freshwater availability and change globally. However, GRACE-TWS records are relatively short for long-term hydroclimatic variability studies, dating back to April 2002. In this paper, we made use of Noah Land Surface Model (LSM), and El Niño–Southern Oscillation (ENSO) data in an autoregressive model with exogenous variables (ARX) to reconstruct a 66-year record of TWS for nine major transboundary river basins (TRBs) in Africa. Model performance was evaluated using standard indicators, including the Nash Sutcliffe Efficiency criteria, cumulative density frequency, standardized residuals plots, and model uncertainty bounds. Temporally, the reconstruction results were evaluated for trend, cycles, and mode of variability against ancillary data from the WaterGAP Model (WGHM-TWS) and GPCC-based precipitation anomalies. The temporal pattern reveals good agreement between the reconstructed TWS, WGHM-TWS, and GPCC, (p-value < 0.0001). The reconstructed TWS suggests a significant declining trend across the northern and central TRBs since 1951, while the southern basins show an insignificant trend. The mode of variability analysis indicates short storage periodicity of four to sixteen-month in the northern basins, while strong intra-annual variability in the central and southern basins. The long-term TWS records provide additional support to Africa’s water resources research on hydroclimatic variability and change in shared transboundary water basins.

## Introduction

Projected dynamics suggest that Africa will confront major challenges on multiple fronts during the next decades that will entail serious adverse impacts on the continent’s water resources^1–5^. Africa’s population, currently estimated at 1.2-Billion^6^, is expected to double, necessitating a significant increase in water supply for sanitation^7^, household use^8^, and food production capacity over the next 30 years^1,9^. Economic activities will expand by 3.7% in 2020–21^10^, accompanied by an exponential increase in energy demand and use, with attendant consequences on water resources^10^. Climate change and climate variability will confound above dynamics, by altering the intensity, magnitude, timing, as well as spatial patterns of water resources availability^1–5^.

To better understand the patterns and effects of climate and environmental changes on Africa’s water resources, data are needed at an increasingly fine spatial and temporal granularity. In reality, however, the observational data networks required to support such analyses have continued to decline in number. In the Nile River Basin, for example, the number of functional hydrometeorological gauging stations declined by an astonishing 88% between the early 1960s and 2014^11^. The situation is similar in nearly all of Africa’s Transboundary River Basins (TRBs)^12,13^. In addition to the question of network density, issues related to data access, quality, temporal continuity, spatial representativeness, and tedious bureaucratic processes, have combined to force researchers investigating Africa’s water resources dynamics to rely increasingly on modeled and satellite data^14–22^.

One source of satellite-based data that has found broad applicability in terrestrial hydrologic research is total water storage anomalies (TWSA) obtained from the Gravity Recovery and Climate Experiment (GRACE) satellites^2,20,23–25^. GRACE-derived TWS data integrate storage changes in all forms of water, including surface water storage (SWS), soil moisture storage (SMS), groundwater storage (GWS), and snow water equivalent (SWE), as well as the impacts of anthropogenic processes on these stocks of water. For data-scarce regions, such as Africa, GRACE TWSA are especially advantageous because the data are publicly accessible, homogenously processed, and derived using the same consistent standards and methodology over time and space. Furthermore, because GRACE-derived TWS reflects the totality of the effects of natural and anthropogenic processes on water resources, the resulting spatial and temporal water storage patterns are unique and distinct from the patterns generated by other combinations of hydroclimatic data^19^. Despite these obvious advantages, GRACE TWS data suffer the limitation of being relatively short (dating back to only 2002) and therefore, unsuitable for studying long-term terrestrial hydroclimatic variability^26^. Longer TWS time series is desirable to accurately capture the full effects of processes like drought compared to, for example, data based on precipitation, soil moisture, or groundwater alone^27^. Moreover, long-term TWS provide a holistic overview of the hydroclimatic variability and the trends in the available water resources. Meanwhile, TWS is an important boundary condition, as well as key variable in land-atmosphere coupling processes^26,28^. As a result, hydrological models may perform poorly over areas where data necessary for estimating TWS are unavailable^28,29^.

Before GRACE observations became available, estimates of TWS relied on Land Surface Model (LSM) outputs, as well as basin-scale water balance calculations (e.g.,^30^). However, several approaches were developed to reproduce sub-decadal climate-driven TWS variability^31^, 2-D TWS past interannual records^32^, and LSM-based monthly and annual TWS^33,34^. By combining GRACE-TWS data with precipitation and temperature estimates in a statistical model^31^, reconstructed gridded sub-decadal TWS changes for the period 1985 to 2015. The statistically derived TWS data showed robust and satisfactory performance in comparison with model results. In the Amazon Basin^32^, combined GRACE-based TWS spatial patterns with *in situ* river level observations, to reconstruct pre-GRACE TWS patterns for the period 1980 to 2008. Their results, which focused on interannual variability, reproduced spatial features consistent with those obtained using climate models^33^ reconstructed a 65-year long time series of monthly and annual TWS over the Amazon Basin using GRACE-based TWSA data and LSM products. Meanwhile^34^, directly utilized Global Land Data Assimilation System Version 2, (hereafter, GLDAS-2), to identify the spatial patterns of TWS over China from 1948 to 2015. Other examples of pre-GRACE records have been reported over Greenland and Antarctica^35^, Liao river basin, Yangtze, and Beishan basin area in China^36–38^.

In this paper, we made use of Noah LSM (a GLDAS-2 product), and ENSO (El Niño–Southern Oscillation) data in an autoregressive model with exogenous variables (ARX) to reconstruct a 66-year record of TWS for nine major TRBs in Africa, (see section 4.2 about ARX model). The TRBs are Nile, Niger, Chad, Volta, Congo, Zambezi, Okavango, Limpopo, and Orange river basins. Together, these basins represent approximately 60% of Africa’s total land area, support more than 600 million people (>55% of Africa’s population), and provide about 80% of Africa’s total renewable water resources^12^, (Table 1). The TWS reconstruction utilized the mass construction block (mascon) solution provided by the Center for Space Research (CSR-M) at the University of Texas at Austin, USA. Compared to traditional spherical harmonic (SH) solutions, mascon products have a higher signal-to-noise ratio, reduced residual noise, and leakage error^39–41^. The mascon data also can be used directly in most hydrologic applications without the necessity for further processing or empirical filtering^39^. Details of the data and the methodology appear in Section 4. We analyzed the spatial and temporal variability in the reconstructed TWS series and compared the significant features and patterns of the TWS variability against other conventional hydroclimatic series. The reconstructed series complement information from existing conventional data sources, as well as provide unique insights into the long-term spatial patterns and temporal trends about TWS in Africa’s TRBs. The following section contains details about the extended TWS results.

**Table 1:** Basic summary of Africa’s major transboundary river basins.

Basin	Area (km^2^)	Length (Km)	Q (m3/s)	Shared by	Pop (mi) (2015)	Climate
Nile	3,254,555	6,853	2,800	Eritrea, Sudan, Ethiopia, Egypt, Tanzania, Congo DR, Rwanda, South Sudan, Uganda, Burundi, Kenya	300	SA
Congo	4,014,500	4,700	41,200	Angola, Tanzania, Central African Republic, Congo Republic, Congo DR, Rwanda, Gabon, South Sudan, Burundi, Cameroon, Malawi, Zambia	77	H
Niger	2,117,700	4,180	5,590	Mauritania, Algeria, Chad, Guinea, Nigeria, Sierra Leone, Ivory Coast, Mali, Niger, Cameroon, Burkina Faso, Benin	130	SA
Chad	2,434,000	950	1,290	Chad, Libya, Sudan, Central African Republic, Algeria, Niger, Cameroon, Nigeria	30	SH
Volta	407,000	1500	1,200	Ghana, Togo, Ivory Coast, Mali, Burkina Faso, Benin	29	SH
Zambezi	1,390,00	2,570	3,400	Angola, Tanzania, Malawi, Congo DR, Zimbabwe, Namibia, Mozambique, Botswana, Zambia	32	SH
Okavango	530,000	1,700	475	Namibia, Zimbabwe, Angola, Botswana	0.9	SA
Limpopo	415,000	1,750	170	Zimbabwe, Mozambique, Botswana, South Africa	14	SA
Orange	973,000	2,200	365	Lesotho, Namibia, Botswana, South Africa	20	SA

The climate of the river basins is categorized as humid (H), sub-humid (SH), and semiarid (SA).

## Result and Discussion

### ARX statistically simulated TWS

**Figure 1:**
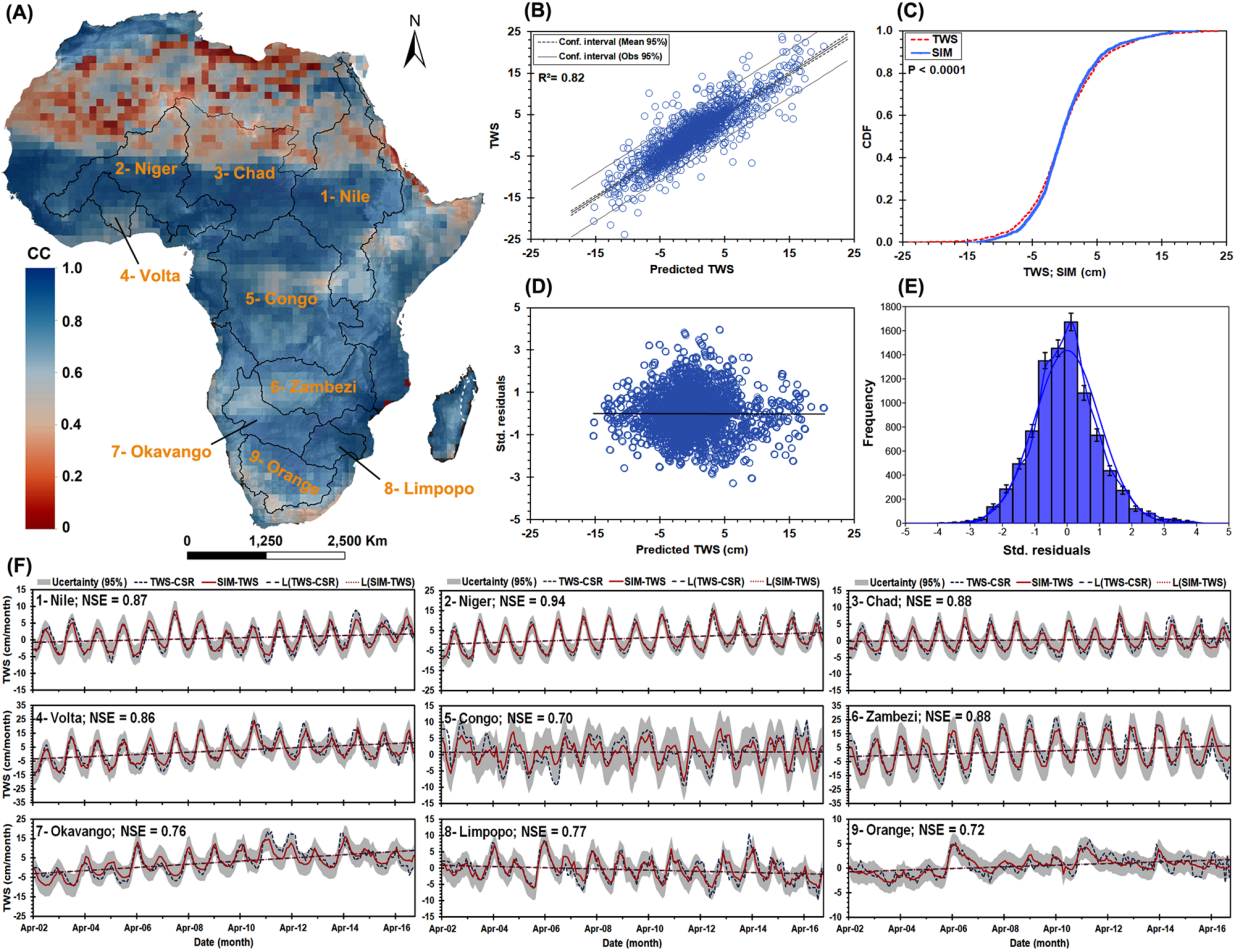
Spatial correlation (**A**) between the simulated TWSA and GRACE CSR-M across Africa between April 2002 to December 2016. The solid black polygons represent the nine studied river basins include (Nile, Niger, Chad, Volta, Congo, Zambezi, Okavango, Limpopo and Orange), see Table 1 for full description. The scatterplot of simulated TWSA and CSR-M across the nine river basins indicates 0.82 correlation (**B**). (**C**) shows the cumulative probability density function (CDF) for the simulated TWSA and CSR-M. (**D**,**E**) show the scatterplot and probability density function (PDF) of the standardized residuals for the nine river basins. (**F**) time series plots show the model performance for the training period for the nine basins with the 95% confidence interval. More details about the model outputs, see SI Figures S1, S2, S7, S8.

**Figure 2:**
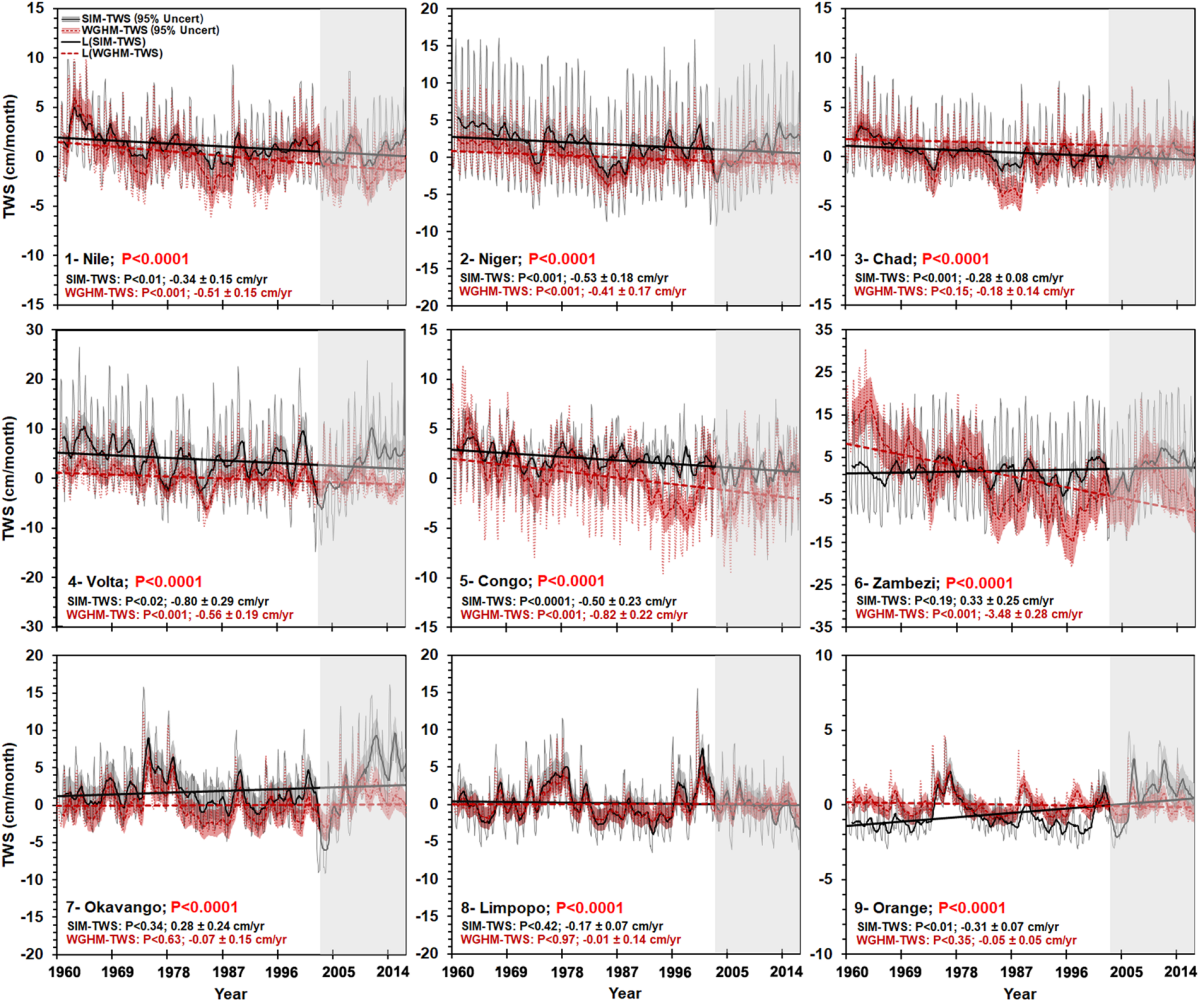
Reconstructed TWS and WGHM-TWS between 1960–2016 (p-value 0.0001), the 12-point moving average with the 95% confidence is represented, the linear trend is indicated using the black and dash-red lines, the shaded gray area represents the period of GRACE-TWS coverage from 2002–2016.

The monthly simulated TWS using ARX model was developed for the period between 1951 to 2016. We evaluated the simulated TWS using standard model evaluation criteria. These include Pearson correlation coefficient (R^2^), cumulative distribution function (CDF), the Nash Sutcliffe efficiency (NSE), Root Mean Square Error (RMSE), goodness-of-fit coefficients (see Table S1), residuals plots, and uncertainty analysis. The uncertainty bounds were calculated according to the approach described in section 4.3. To validate the simulated TWS, we compared the simulated TWS against CSR-M between April 2002 to December 2016; Figure S1 shows the temporal agreement between CSR-M, simulated TWS, and WGHM-TWS. Continent-wide, the correlation (R^2^) between CSR-M and simulated TWS is 0.80 (p < 0.0001). Areas of relatively low agreement (0.50 ≤ R^2^ ≤ 0.60) exist, however, in some locations, notably the Sahara, the semiarid regions in the horn of Africa, and the extreme western part of southern Africa (Fig. 1A). In terms of time, across the nine TRBs, the R^2^ between CSR-M and simulated TWS is 0.82 (p < 0.0001) (Fig. 1B). All evaluation results (Fig. 1C) are satisfactory to excellent. Notably, the standardized residuals are random (Fig. 1D) and follow the normal distribution (Fig. 1E); Additional plots of standardized residuals is indicated in Figure S2. According to^42^, a simulation is considered unsatisfactory if NSE < 0.50, satisfactory if 0.50< NSE <0.65; good, if 0.65< NSE <0.75, and very good if 0.75< NSE <1.0. Based on these thresholds, the simulated TWS across Africa’s TRBs are good to very good model performance (Fig. 1F). The strongest NSE values were found in the Niger, Chad, Zambezi, Nile, and Volta river basins, all of which encompass substantial semiarid conditions. The lowest NSEs were in the Congo and the Orange river basins. As further verification, we compared the reconstructed TWS for each TRB to the modeled TWS derived from the WaterGAP Global Hydrology Model data (WGHM-TWS) between 1960 and 2016^43^, (see, Fig. 2). The results show excellent agreement (p < 0.0001) across all TRBs except for the Zambezi river basin, which shows some discrepancies in the secular (long-term) trend; further research is required to understand the sources for such discrepancies over Zambezi river basin.

### TWS spatiotemporal variation

**Figure 3:**
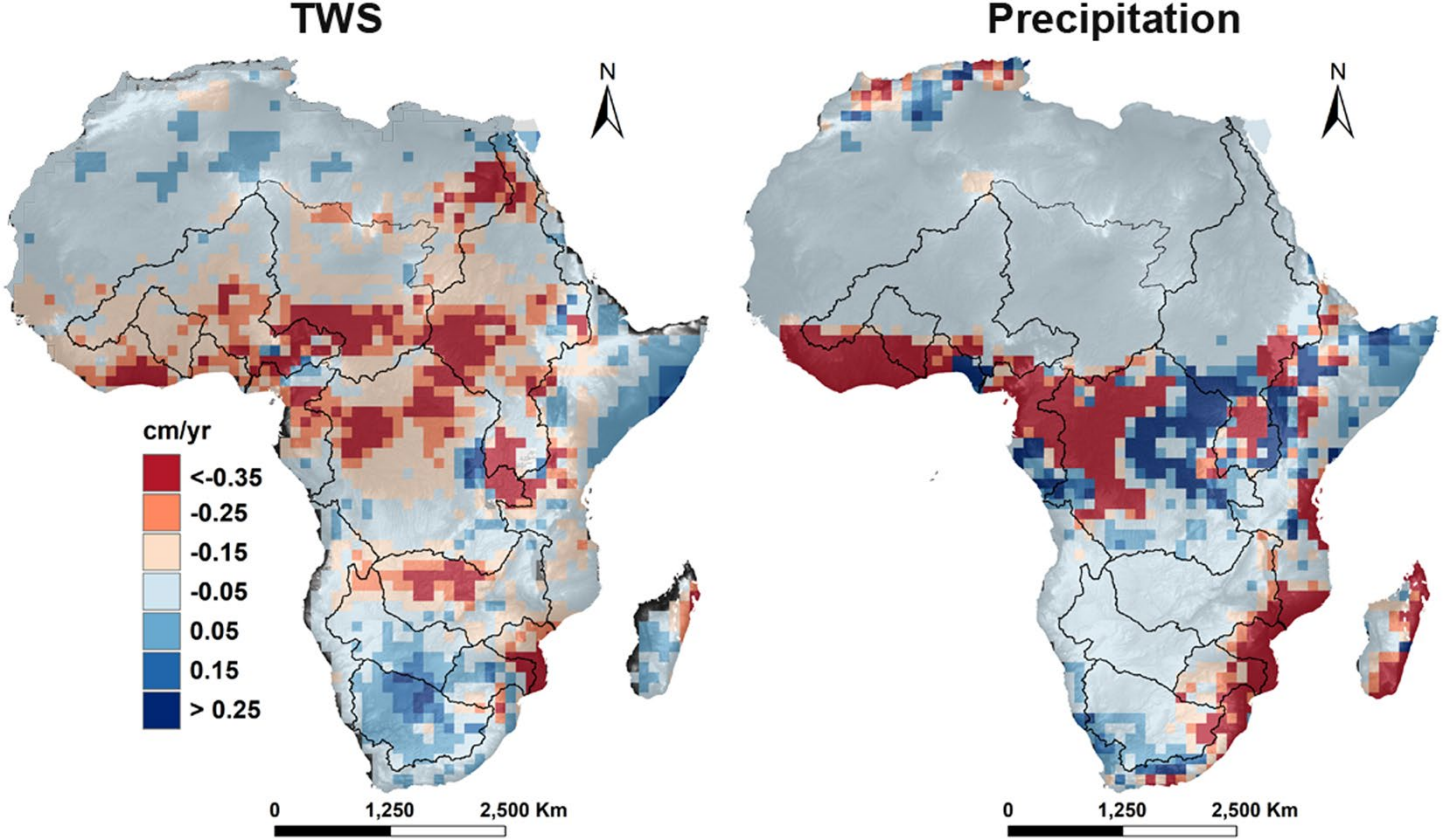
Spatial variation of the reconstructed TWS and precipitation trends across Africa from 1951 to 2016.

On the strength of the agreement between observed and modeled TWS, we reconstructed the monthly TWS at pixel scale across Africa from 1951 to 2016 and examined the trends (Fig. 3). The most prominent features of Fig. 3 are *hotspots*, where TWS trends exceed ± 0.20 cm/yr. over the study period. The positive hotspots indicate areas of upward TWS trend, while negative hotspots represent downward TWS trend. These hotspots are notable because they show a pattern unlike any observed using other hydroclimatic data sets, e.g., precipitation estimates from the same period (see Fig. 3). However, the negative hotspots in the Sahel area show relative agreement to precipitation trend. Spatially, negative hotspots appear concentrated across the interior and central parts of Africa, lower Nile basin, Nubian Aquifer, lower Chad and Niger basin, Central Congo, Upper Zambezi, and southern coastal Mozambique. Positive hotspots prevail along coastal Somalia, the Okavango Delta, and the eastern Democratic Republic of Congo. We theorize that these hotspots reflect the integrated effects of geophysical and anthropogenic activities, notably land use changes. However, further research is needed to confirm their specific locations and extent as well as provide explanations for their existence.

**Figure 4:**
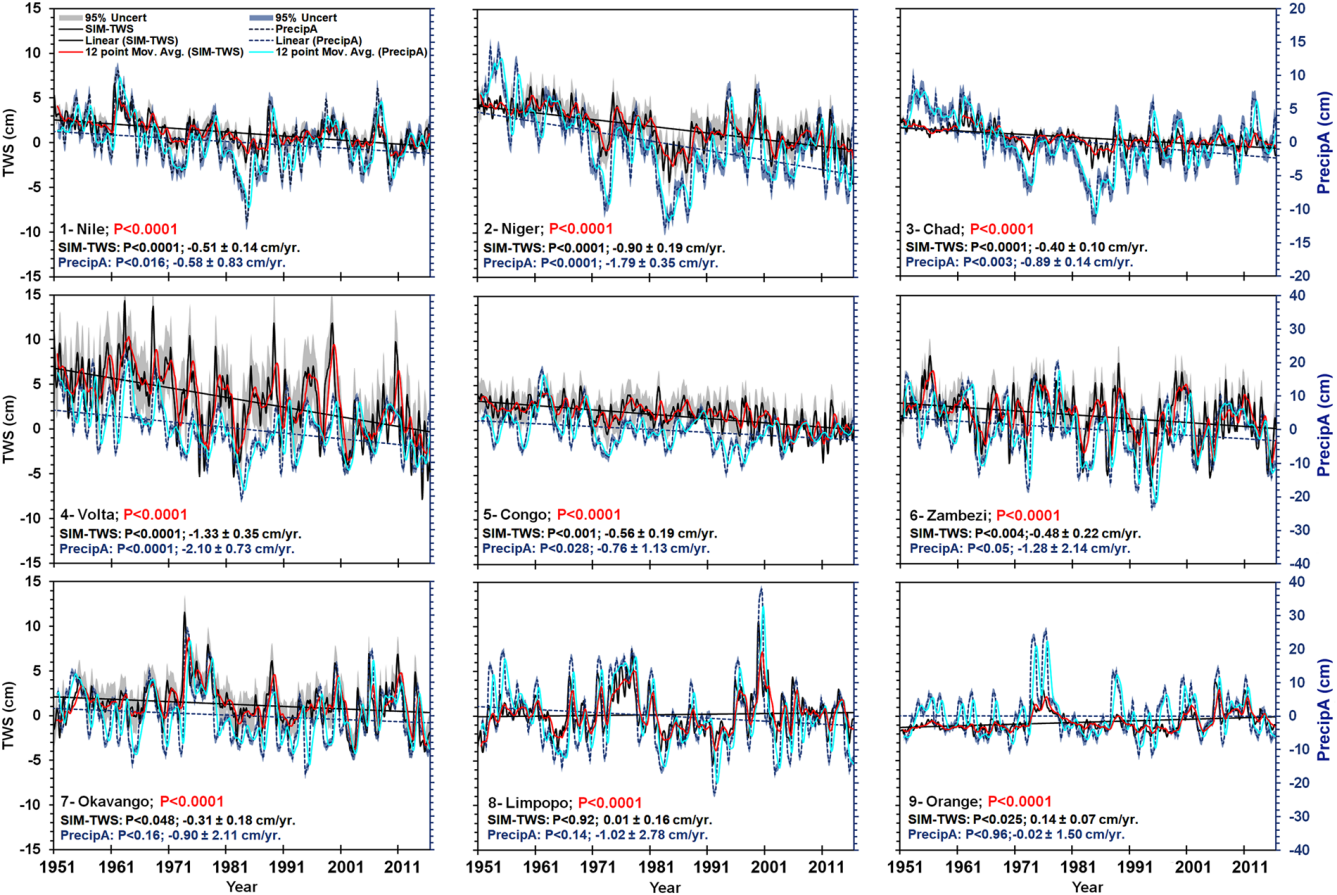
Trend and cyclic components of the reconstructed TWSA and precipitation anomalies, the p-value indicates a good temporal agreement between precipitation anomalies and TWS, the seasonal analysis indicate a one-lag between the precipitation anomaly and TWSA.

Temporally, Fig. 4 compares the cyclical pattern of reconstructed TWSA between 1951 and 2016 with precipitation anomalies derived from the Global Precipitation Climatology Centre (GPCC). The precipitation anomalies show strong temporal coevolution with the one month lagged TWSA (p-value < 0.001). Except for the Limpopo and Orange basins, all of the studied basins showed declining TWSA time series from 1951. However, three general patterns of temporal TWSA trends are discernible. The first is statistically significant (p < 0.0001) downward trend in the northern (Nile, Niger, Chad, Volta) and central (Congo, Zambezi, and Okavango) basins. In terms of magnitude, the declines ranged between − 0.31 cm ± 0.18 cm/year in Okavango Basin and − 1.33 cm ± 0.35 cm/year in the Volta basin. The second pattern is a slight upward but statistically insignificant trend in the Limpopo river basin. Finally, the Orange basin experienced significant (p < 0.001) upward trend of 0.14 cm ± 0.07 cm/year during the study period. Additionally, some of the basins (e.g. Volta, Zambezi) display strong intra-annual variability while others (e.g. Nile, Chad, and Limpopo), are characterized by strong runs. Finally, as might be expected, the variability in the precipitation anomalies is greater than TWSA.

### Wavelet analysis

**Figure 5:**
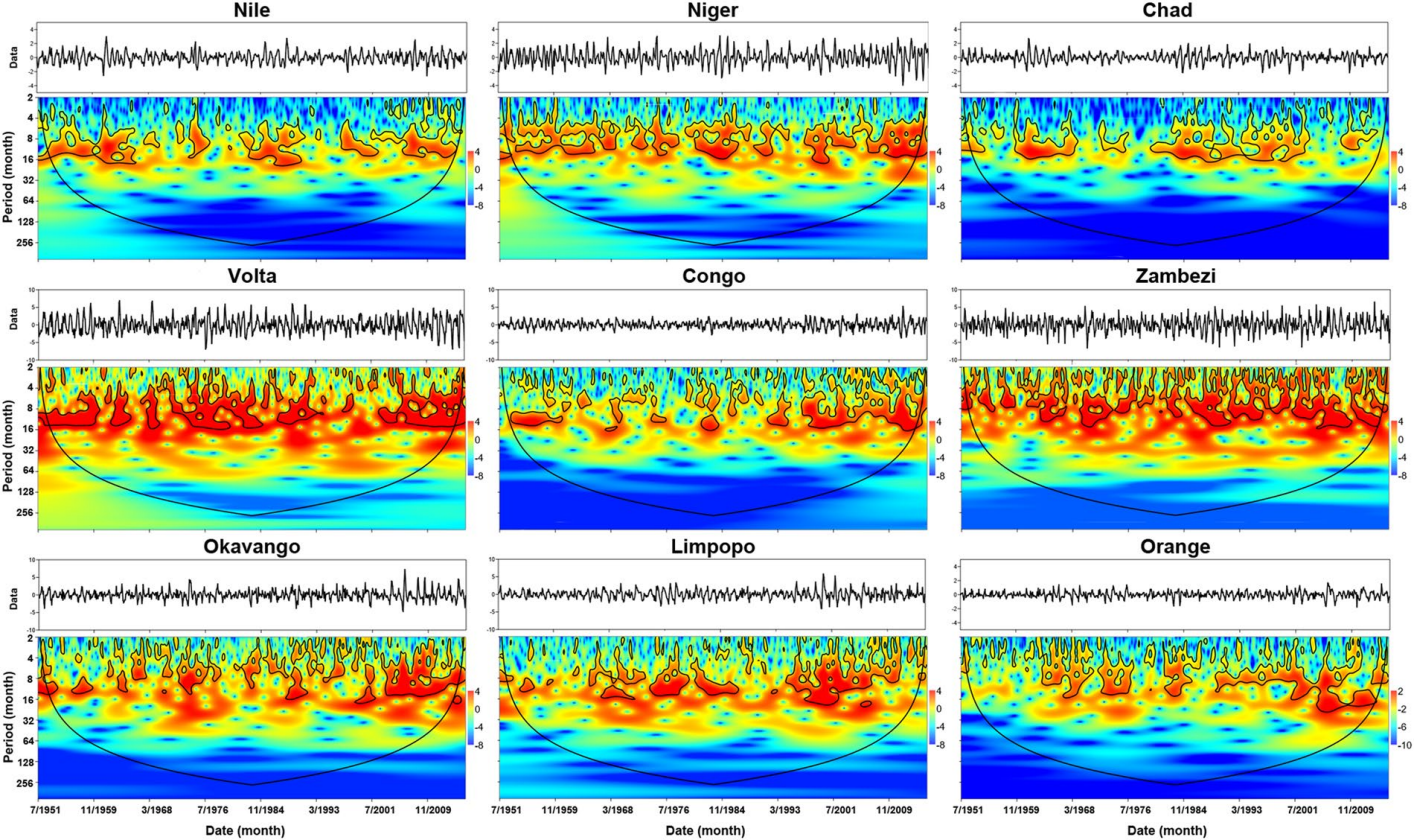
The continuous wavelet power spectrum of the reconstructed TWSA for the nine river basins.

Figure 5 shows the dominant modes of variability in the reconstructed TWS, using wavelet power spectra (see section 4.4). The plot shows areas of significant (α < 0.05) power variability enclosed by bold isolines against a red noise background. The marked cone of influence, beyond which edge effects may compromise the validity of interpretations. Following conventional practice, we ignored this portion of the plot in our interpretation. The power spectrums suggest that high and moderate frequency modes of variability (sub-annual to cycles of 4 to 16-month) dominate the TWS series across the TRBs basins. Cycles on the order of 16-month are also discernible but much less prominent. The plots show low to moderate periodicities in the northern basins with a possible change in hydroclimatic regime, indicated by strong intra-annual variability starting from late 2000s, except for the Chad river basin. The Volta basin shows three types of shifts in wavelet power; between 1951 to 1959, 1977 and 1980, and finally, an increase in wavelet activity after 2010.

Contrastingly, the southern basins (Zambezi, Okavango, Limpopo, and Orange) are characterized by periodicities with strong intra-annual variability. The Zambezi river basin shows constant periodicities with substantial intra-annual variability. The Okavango basin shows strong power spectrum starting in early 1990 and became stronger starting early 2000s. The Limpopo river basin shows two periods of intense power spectrum, one from 1969 to 1984, and the second from early 1994 to 2016. The Orange Basin stands out as the only one with continuous power variability from 1960 to 2016 of strong sub-annual of 8-month cycles with a high amplitude between 1968 and 1977; after 1998, strong 16-month cycles become dominant. The Orange Basin shows significant power spectrum after 1994.

Beyond the above broad patterns, the TWS also varies in ways that are unique to each basin, reflecting local hydroclimatic dynamics. In the Nile basin, for example, three periods of significant wavelet activity stand out, in the 1950s, between 1984 and 1990, and after 1997. Inferences from other independent sources of data, e.g., precipitation, suggest that the variability of power in the between 1984 and 1991 corresponds to a basin-wide drought episode with enhanced wavelet activity after 1997. Similarly, in the Chad Basin, there are three distinct periods of wavelet power variability (1950s-1972, 1984–1995, and after 2000), all of which accord with episodes of major hydroclimatic shocks that are well documented in other sources^44^. The Chad Basin, experiences relatively small amplitudes of power variability, indicating less dramatic changes in basin water storage. On the whole, the evidence points to a significant shift in the hydroclimatic regime that has been ongoing in the northern basins since the early 1990s. The southern basins, however, shows constant wavelet activity with strong seasonal cycles and possible regime shift in power during the early 2000s.

### Summary and conclusion

This paper contributes to incipient efforts to reconstruct TWSA data for Africa’s TRBs. The nine studied TRBs are shared by between four and thirteen countries, all of which are experiencing similar dynamics, namely, rapid population growth, natural and anthropogenic environmental change, climate change, and climate variability. The TWS of the TRBs integrate the effects of all of the above dynamics and therefore necessitate closer monitoring than ever before. Regrettably, the observational hydro-climatological networks have diminished in areal coverage, and operational capabilities have become degraded. TWS data measured by GRACE satellite are uniquely suited to this challenge because the data are continuous over time and space and easy to access. A persisting limitation of the GRACE data, however, is the short period coverage (since 2002) with a gap in data after late 2017, which constrains its use for investigating long-term hydroclimatic changes. In this paper, therefore, we utilized Noah LSM and ENSO data in the framework of an ARX modeling approach to statistically simulate TWSA based on GRACE observational data for the period between 2002 to 2016. The simulated data agreed strongly both temporally and spatially with the GRACE CSR-M data as evaluated by the Pearson correlation coefficient, CDF, NSE coefficient, and model uncertainty. The LSM and ENSO are driven ARX multivariate regression results that explain 82% of the temporal variability in GRACE CSR-M over the calibration period. Spatially, the results contain areas of relatively low agreement between simulated and reconstructed TWSA. The results of reconstructed TWSA in these regions can be expected to have broader or larger uncertainty bounds and should, therefore, be interpreted and used cautiously.

On the strength of the agreement achieved, we reconstructed a 66-years of extended TWS data for Africa’s major TRBs and investigated the dominant features and characteristics of the hydroclimatic variability over space and time. Similar to the calibration period, the reconstructed TWS grids identified coherent zones of elevated negative and positive trends or hotspots. The spatial distribution and patterns of hotspots occur exclusively in the TWS series, i.e., they are not reproduced in other hydroclimatic time series. This unique perspective likely reflects the fact that, at each grid point, the TWS integrates all of the columnar changes in surface water, soil moisture, and groundwater arising from both natural processes and anthropogenic activities. The areas of negative trends occur throughout the internal parts of the continent and vastly outnumber areas with increasing trends. The areas of positive trends are remarkable for their locations, including the arid regions of Somalia and the horn of Africa and the region around the Okavango Delta. Again, additional research is needed both to establish the robustness of these patterns as well as the reasons for their occurrence.

The results of wavelet analysis show that the dominant mode of variability in the TWS time series are high-frequency events on the scale of sub-annual to about 4 or 16-month periodicities. No decadal scale periodicities were detected. The study did not attempt to compare these variabilities with those found in other hydroclimatic time series. We suspect that the same analysis conducted on annual data series would probably yield different conclusions.

While the reconstructed TWSA revealed new features not previously seen in other hydroclimatic series, it also generally reproduced many of the features that researchers have described in detail in other conventional hydroclimatic data series in Africa. These include, for example, declining trends in the time series of rainfall. This concordance lends credibility to the validity and robustness of the TWSA reconstructed series. Further studies are required to explain both the areas of poor agreement between modeled and simulated TWSA, as well as the seemingly unique features suggested by the TWS. In general, however, the reconstructed TWS are robust and can be used as an independent/additional source of data for studying water resources dynamics in Africa. TWS has been declining across all of Africa’s major TRBs since 1951. This decline manifests as a gradual trend in the TWS time series in each of the nine river basins. Such decline may have implications on total water resources availability and use, as well as the rules or accords underpinning shared water use by the countries of the basins.

## Materials and Methods

### GRACE, LSMs and precipitation data

The original gridded observations of GRACE data used to study regional, and basin-scale changes in TWS were developed via Spherical Harmonics (SH) solution^45–49^. These SH solutions suffered signal attenuation and leakage that required a scaling factor to reduce noise and amplify the signals^23,50^. To mitigate these effects, GRACE mass concentration blocks or (mascon) were introduced. However, due to the original GRACE footprint, the spatial resolution of GRACE data is around 300 km, either using SH or mascon solutions. Meanwhile, the mascon solutions were first developed by the gravity group at National Aeronautics and Space Administration (NASA) Goddard Space Flight Center (GSFC), (GSFC-M)^51,52^. In 2015, NASA Jet Propulsion Laboratory (JPL) introduced the (JPL-M) that estimated the TWSA at 3-degree grids^41^. The JPL-M, however, requires the application a scaling factor to retain the TWS signals to smaller domains. In 2016, the Center for Space Research (CSR) at the University of Texas at Austin (UT, Austin) introduced another mascons product (CSR-M) that were estimated using the same standards as the preceding SH^40^. The CSR-M solutions use an equal area grid with hexagonal tiles ~120 km wide at the equator^39,40^. The new CSR-M solutions, however, require no preprocessing or the utilization of a scaling factor for signals restoration. The CSR-M solutions can be used directly for any hydrologic application^39,40,53^. The CSR data were extensively utilized in assessing hydrologic extremes^54^, decadal groundwater changes^55,56^, and water scarcity^57^, among other hydrological applications^57,58^. The CSR-M data are available at http://www2.csr.utexas.edu/grace/RL05_mascons.html.

The Noah Land Surface Model (LSM) estimates from 1951 to 2016 data, a product of GLDAS-2 that integrate a large number of observation-based data to drive multiple LSMs^59,60^. The GLDAS-2 model was forced using consistent global meteorological, forcing datasets from Princeton University^59^. The Princeton Global Forcing variables extended from 1948 to 2016, and include precipitation, air temperature, downward longwave, downward shortwave, surface pressure, specific humidity, and wind speed. The Princeton forcing variables were subjected to several updates that eliminate any spurious values or unrealistic estimates^61^. An additional enhancement to GLDAS-2 includes the switching to MODIS-based land surface parameters and initialization of soil moisture over deserts. Each simulation used the common GLDAS dataset for land water mask (MOD44W^62^:), soil texture^63^, and elevation (GTOPO30) along with MODIS default landcover datasets (MCD12Q1^64^:). More details regarding the LSM parameters at https://ldas.gsfc.nasa.gov/gldas/.

Noah-LSM has been utilized extensively to estimate equivalent land water content (LWC), which integrates soil moisture at four depths (0:10, 10:40, 40:100 and 100:200 cm below ground surface), as well as the canopy water content (CWC)^60,65^. Herein, five LWC constituents from Noah-LSM data were incorporated along with ENSO signals (climate variable) to reproduce the long-term TWS estimates for Africa’s major TRBs between 1951 to 2016. Noah-LSM is available in two versions (V2.0 from 1948 to 2010 and V2.1 from 2000 to 2018). We utilized the monthly time step Noah-LSM data at a 0.25-degree resolution grid; the data were scaled up to 0.5-degree. GLDAS Noah-LSM data can be accessed via https://disc.sci.gsfc.nasa.gov/datasets.

The WaterGAP Global Hydrology Model V2, WGHM, produced globally at 0.5-degree resolution grids, and provide the total water storage estimates^43^. For this study, we derived the WGHM-TWS from 1960 to 2016 by subtracting the long-term mean average from each monthly gridded observations. Precipitation anomaly data were derived using a 0.5-degree resolution gauge-corrected precipitation data from the Deutscher Wetterdienst (DWD) center in Germany (http://gpcc.dwd.de)^66^. We followed the same approach used to calculate the WGHM-TWS, i.e., by subtracting the long-term mean average for the entire study period from each monthly data point. The uncertainties associated with each dataset in this research were evaluated according to the approach described in section 4.3.

El Niño and La Niña together called the El Niño Southern Oscillation (ENSO), describe the periodic departures of sea surface temperatures (SSTs) from normal across the Equatorial Pacific Ocean. ENSO affects weather patterns worldwide and may intensify moisture advection, and therefore precipitation, to some regions while inducing extremes droughts in others^67,68^. ENSO data are available via the Earth System Research Laboratory (ESRL) portal at https://www.esrl.noaa.gov/psd/enso/.

### Statistical simulation

Noah LSM modeling generates outputs of terrestrial storage comparable to GRACE TWS data^2,24,34,48,57,60,69,70^. The difference between the two is that GRACE-TWS comprises changes in all forms of water storage, including SWS, while LSM data lack the GWS component (see, Figures S3, S4), i.e.;

1 $$\Delta GWS=\Delta TWS-\Delta SMS$$

Because TWS comprises storage changes from several variables and processes, multivariate regression modeling can be utilized to explain the variability in TWS due to the contributions and interactions of the known independent variables or regressors (see Figure S5). For this study, we made use of an autoregressive multivariate statistical approach with exogenous variables (ARX) first to capture the variability in GRACE-TWS over nine TRBs in Africa and then reconstructed the TWS for each TRB back to 1951. The regressor variables included four SMS layers and CWC. We ignored the SWE since the vast majority of Africa receives no snowfall (see Figure S6). For more in-depth analysis at specific locations, such as the Atlas Mountains or the Highlands of East and Southern Africa, however, it may be useful to include the SWE.

The analytic process comprised the following steps: First, the predicted TWS (CSR-M) and predictor variables (four SMS layers, CWC, and the ENSO signals) were decomposed using a linear regression approach into a trend and stochastic (detrended) components. Next, for both trend and random stochastic series, we reproduced a training dataset of 1000-random points using a random sample technique without replacement from the training time series between 2002 to 2016; The sample tool is available via CARN.R-project, sampling survey package^71^. Third, a two-step ARX model was applied, the ARX function is available via CRAN.R-project, simulation, estimation, model selection, and forecasting package^72^. The ARX model in which exogenous multiple inputs, (predictors), are used to generate single output, (predicted), (see Figure S7, S8), is following a system of linear equations to capture the relationship between two consecutive values of the system outputs and inputs^28,73^, described as:

2 $${Y}_{t}={\varepsilon }_{t}+\mathop{\sum }\limits_{i=1}^{p}{a}_{i}{Y}_{t-i}+\mathop{\sum }\limits_{i=1}^{q}{m}_{i}{{\rm{\varepsilon }}}_{t-i}+\mathop{\sum }\limits_{i=1}^{b}{n}_{i}{{\rm{d}}}_{t-i}$$

where *Y*_*t*_ is the independent TWSA; (*p, q, b*) represent, respectively, autoregressive, *p*, moving average, *q*, and exogenous inputs terms, *b*; (*a*_*i*_, *m*_*i*_, *n*_*i*_) represent the model coefficient for seasonal, moving average and exogenous terms; the error term (*ε*_*t*_) are generally assumed to be independent and randomly distributed. ARX model performance is presented in section 4.3.

**Figure 6:**
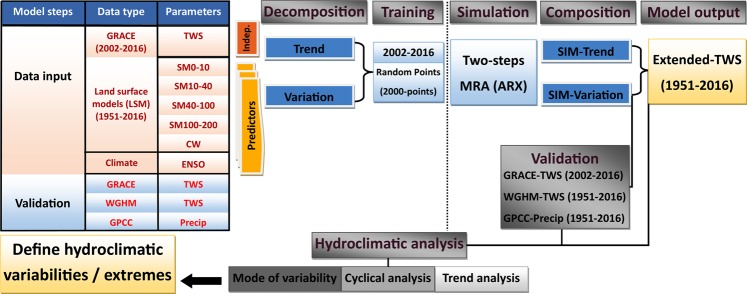
The workflow of the data analysis and TWS reconstruction.

Fourth, to fit an ARX model, the ordinary least squares method (OLS) was used in which the sum of squared errors ${\varepsilon }_{i}^{2}$ is minimized, provides an exact analytical solution, where, the *Y*_*t*_ is the expression of random variables with mean *μ*_*i*_ and variance σ^2^. Finally, the ARX model outputs for trend and stochastic component were coupled back to produce a total simulated TWS series. Figure 6 shows a conceptual framework of two-steps ARX statistical approach and TWS analysis.

### Model performance, trend, and uncertainty analysis

Figure 1F shows the reconstructed time series for the nine river basins. The simulated TWS data are evaluated against the reference CSR-M using Pearson correlation coefficient, the CDF distribution, and the Nash-Sutcliffe (NSE) coefficient. The 95% two-sided confidence interval was calculated around the mean (μ) of reference and simulated TWS; the confidence levels enclose all acceptable values within (1 − α; α = 0.5).

The secular trend (long-term trend) in GRACE-derived TWS, simulated TWS, and WGHM-TWS were extracted by simultaneously fitting a trend and a seasonal term to teach time series. Uncertainties associated with the calculated trend values from each series were estimated according to^39,74^. The residuals (R1), were calculated after removing the deterministic components (long-term trend, annual, and semi-annual components). Then, the interannual signals were removed from (R1) by fitting a 13-month moving average; a linear regression approach was applied to remove any further trend signals, then a second residual series, (R2), was calculated. The standard deviation of the residual (R2) was interpreted as maximum uncertainty or measurement error (the amplitude of the measurement error)^39,56,74,75^.

### Analysis of TWS variability

As with any time series, the long-term TWSA contains multiple features and modes of variability, such as long-term trends, cyclicity, seasonality, and stochastic (residual) components.

3 $${S}_{total}=({S}_{trend}+{S}_{cycle})+{S}_{seasonal}+{S}_{residual}$$

It is crucial to identify the occurrence and magnitudes of these features or component in the time series components^76^. From a practical perspective, the variabilities characterizing a time series have implications for planning in different sectors and activities^77^. From a research perspective, decomposing a time series into its constituent variability modes provides insight into the causal processes driving the system.

The reconstructed TWSA times series of all of Africa’s contain strong cyclical components. Therefore, we first applied a linear regression approach to isolate linear trend then we used an additive decomposition approach to remove the interannual variability and seasonal pattern to produce a white-noise stochastic series for each river basin. Then the residual term was analyzed further and a linear regression approach was applied to remove any remaining trend. These processes produced a stochastic (white-noise) time series, which was then analyzed using a continuous wavelet transform (CWT) tool. Unlike the Fourier transform (FT), which only breaks down a time series signal into sinusoids of different frequencies. The CWT decomposes a time series into frequency-time space, making it possible to determine the dominant modes of variability and the evolution of those modes in time^78^. For a given time series, *x(t)*, with equal sampling time interval, *δt*, the CWT is the convolution of *x(t)* with scaled and shifted versions of a mother wavelet *ψ*(*τ*). Mathematically, the CWT is expressed as:

4 $$C(\eta ,s)={s}^{-1/2}{\int }_{-\infty }^{+\infty }x(t){\psi }^{\ast }(\frac{t-\eta }{s})\delta t$$

where, C(η,*s*) are the CWT coefficients, and η, *s* are the scale and the shift (time) parameters respectively. The symbol (*) denotes the complex conjugate. The resulting wavelet coefficients are used to produce the wavelet power spectrum, traditionally defined as ${|C(\eta ,s)|}^{2}$. This gives the fluctuation of the variance across different frequencies and over time in a 2-dimensional scale/frequency-time plot. For geophysical and hydroclimatic data, the mother wavelet commonly used is the Morlet, which is the product (point by point) of a sine wave and the Gaussian. It has the mathematical form:

5 $$\psi (\tau )={\pi }^{-0.25}{e}^{i{\omega }_{0}\tau }{e}^{-{\tau }^{2}/2}$$

where *ψ* is a wavelet function dependent on the non-dimensional time parameter τ, ω0 is the non-dimensional frequency, and *i* = √−1. Because the Morlet wavelet is a complex function, it returns both amplitude and phase information when applied to a time series signal, making it uniquely suited for capturing oscillatory behavior^77,79^. For this study, the Morlet wavelet was utilized to perform CWT on TWSA time series for each transboundary river basin. The analyses were accomplished in a MATLAB platform using a modified version of the code provided by^78^. This modification is based on the work of^80^, who argued that the traditional bias toward larger scales in the wavelet power spectrum still exists in the Torrence and Compo code^78^. The authors attributed the bias to the wrong definition of the “energy” for the wavelet power spectra, which is taken as the square of the wavelet coefficients that be normalized by the associated scales^80^.

## Supplementary information

Supplementary info

## Data Availability

Data utilized in this research and the simulated long-term TWS can be requested by contacting the first author at a reasonable request.
