# Supplementary material for: +50 Years of Terrestrial Hydroclimatic Variability in Africa’s Transboundary Waters
Source: Sci Rep. 2019 Aug 23;9:12327. doi: 10.1038/s41598-019-48813-x (PMC6707189; doi:10.1038/s41598-019-48813-x)
Supplement: Supplementary file 1 — Supplementary info [file 41598_2019_48813_MOESM1_ESM.docx]

**Supplementary Information for**

**+50 Years of Terrestrial Hydroclimatic Variability in Africa’s Transboundary Waters**

Emad Hasan^1,3,7*,^ Aondover Tarhule^1^, Joseph T. Zume^2^, Pierre-Emmanuel Kirstetter^4,5,6^

^1^Department of Geography, State University of New York, SUNY at Binghamton, NY, USA.

^2^Geography and Earth Science Department, Shippensburg University, Shippensburg, PA, USA.

^3^Hydrometrology and Remote Sensing (HyDROS) laboratory, University of Oklahoma, Norman, OK, USA.

^4^School of Meteorology, University of Oklahoma, Norman, OK

^5^School of Civil Engineering and Environmental Science, University of Oklahoma, Norman, OK

^6^NOAA/National Severe Storms Laboratory, Norman, OK

^7^Geology Department, Faculty of Science, Damietta University, New Damietta, Egypt.

**Contents of this file**

Figures S1 to S8

Table S1


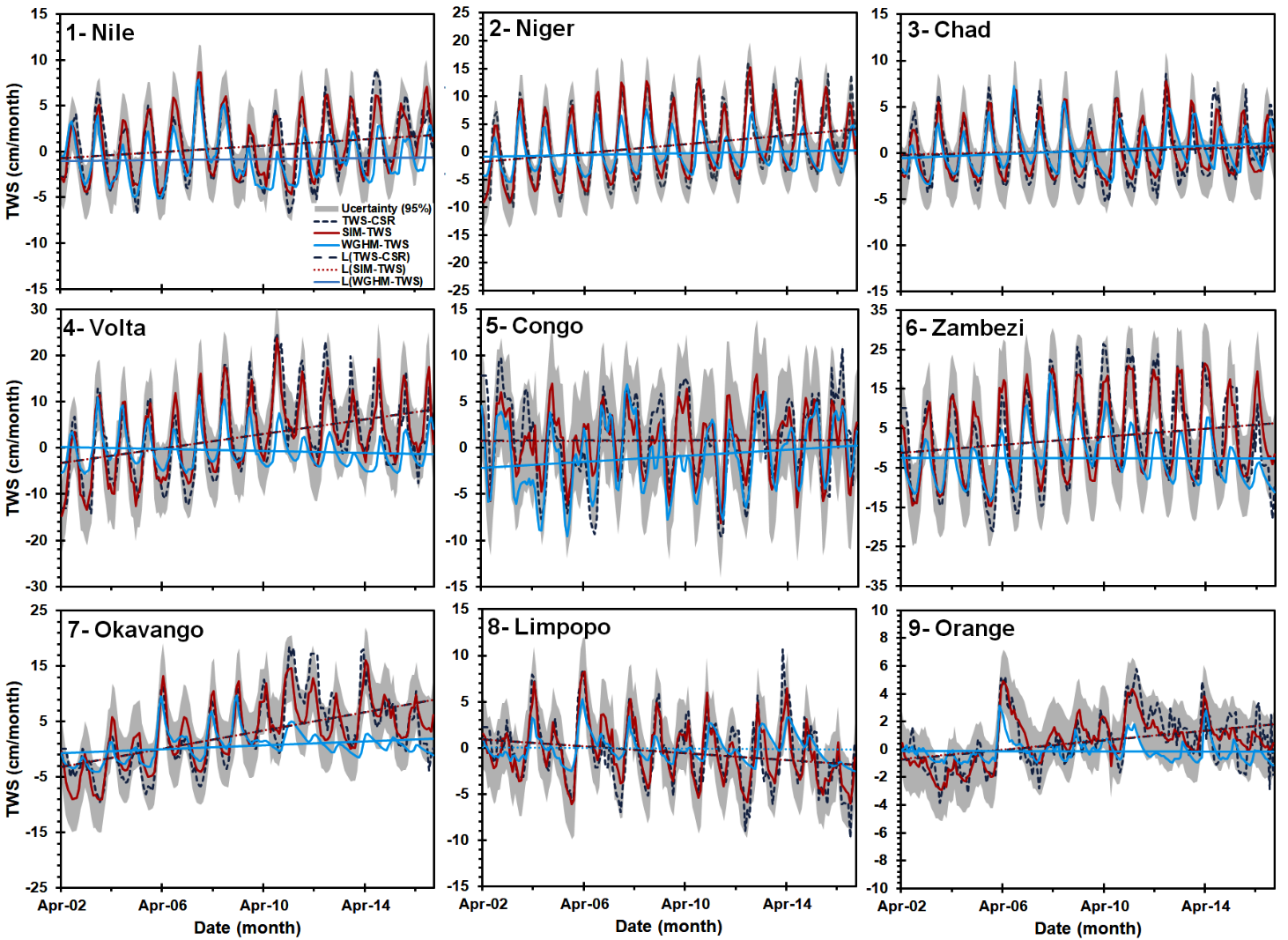


Figure S1. Temporal agreement between GRACE-TWS (block-dotted line), Simulated TWS (solid-red line), and WGHM-TWS (Solid-cyan line) from 2002 and 2016. The shaded gray areas represent the 95 percent uncertainty/confidence around SIM-TWS. The linear trend (L) of GRACE-TWS and SIM-TWS show a very agreement relative to the linear trend of WGHM-TWS. Compared to Figure S4, the ARX model was applied to eliminate the recent divergence between CSR-M observations and simulated TWS-based on Noah-LSM model observation.


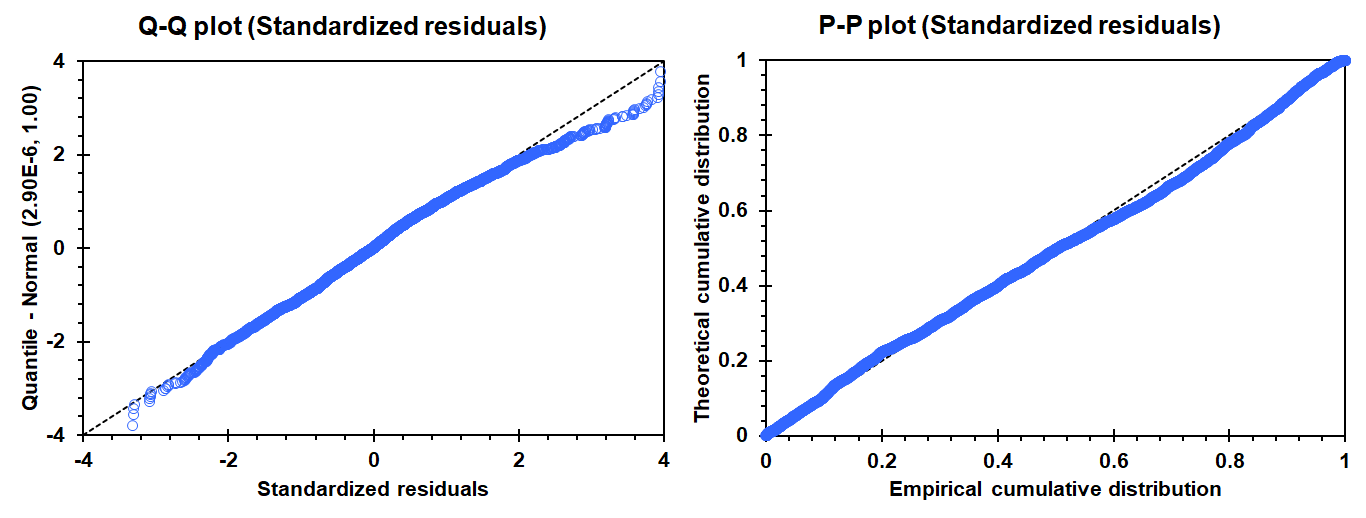
Figure S2. Q-Q and P-P plots of standardized residuals of the SIM-TWS for the nine river basins.


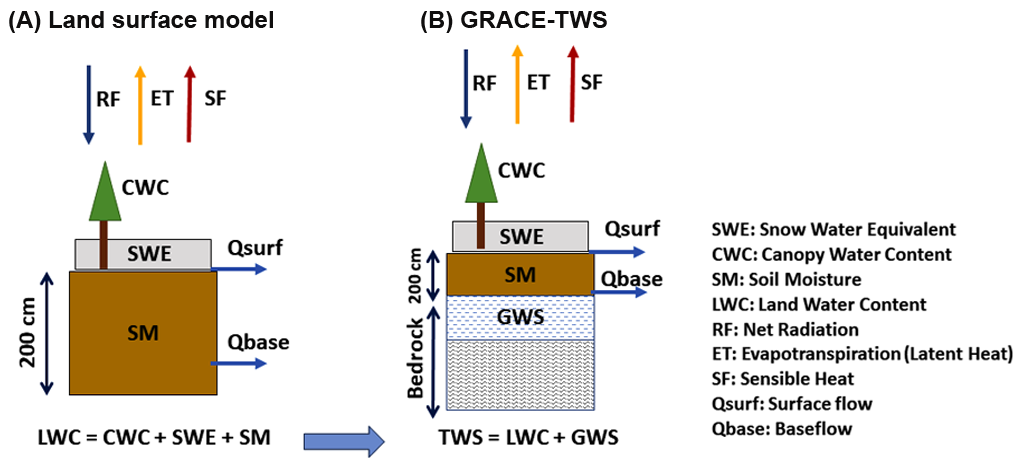


Figure S3. Illustration for the storage component derived from LSM (A), and GRACE-TWS (B). GRACE satellite does not distinguish different storage component. Therefore, LSM observations were utilized to isolate different component from GRACE, especially groundwater observation. It is noteworthy that Noah-LSM version 2 was forced using global meteorological forcing data set from Princeton University, MODIS land cover (MCD12Q1), land-water mask (MOD44W), soil texture, and elevation data (GTOPO30).


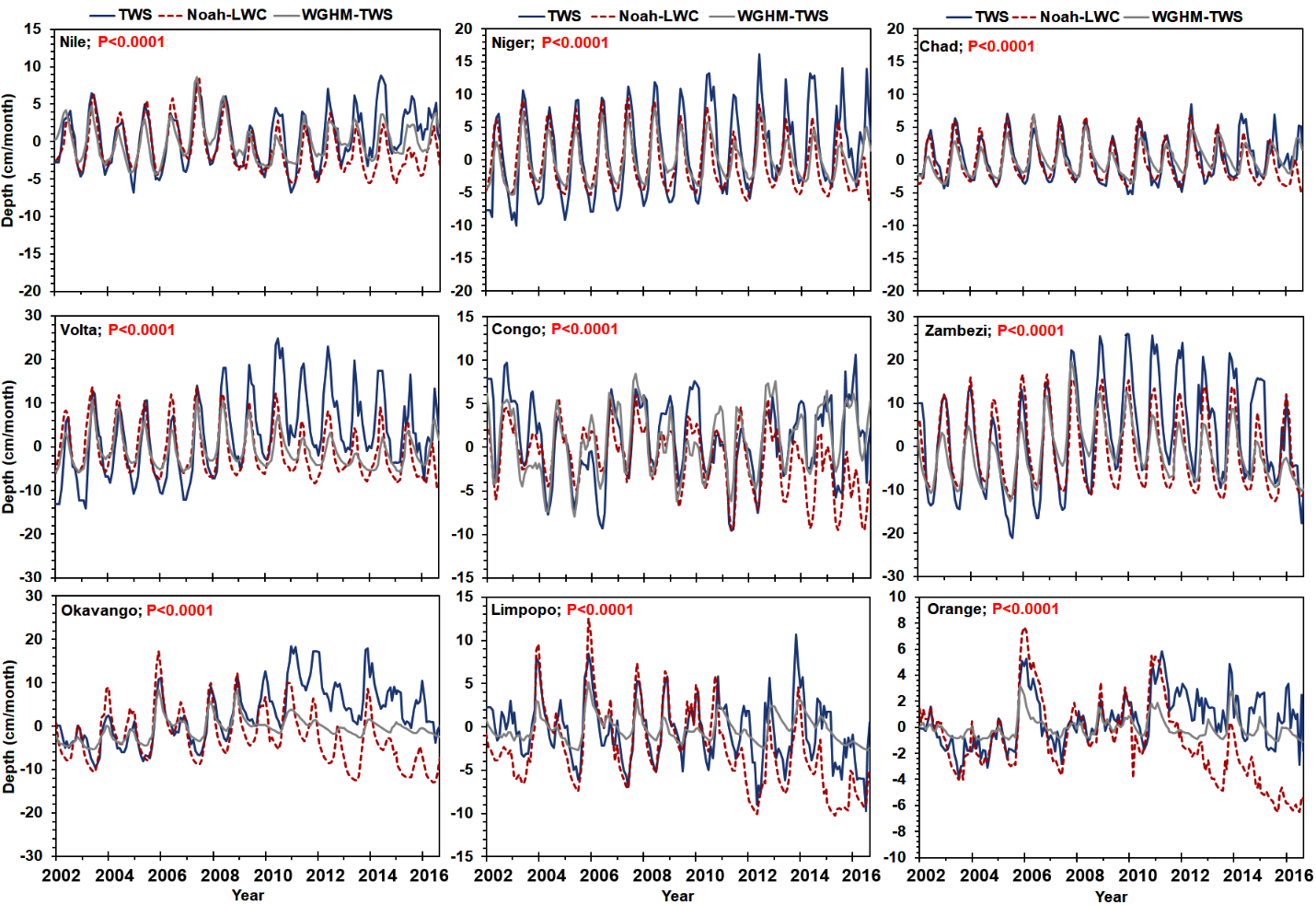


Figure S4. Temporal agreement between CSR-M TWS, Noah-LSM LWC, and WGHM-TWS, the p-value indicates a good agreement between storage components. Note the divergence in trend between the CSR-M, LWC, and WGHM-TWS from 2010 to 2016, especially, in the southern basins; further research is needed to understand the nature of such divergence.


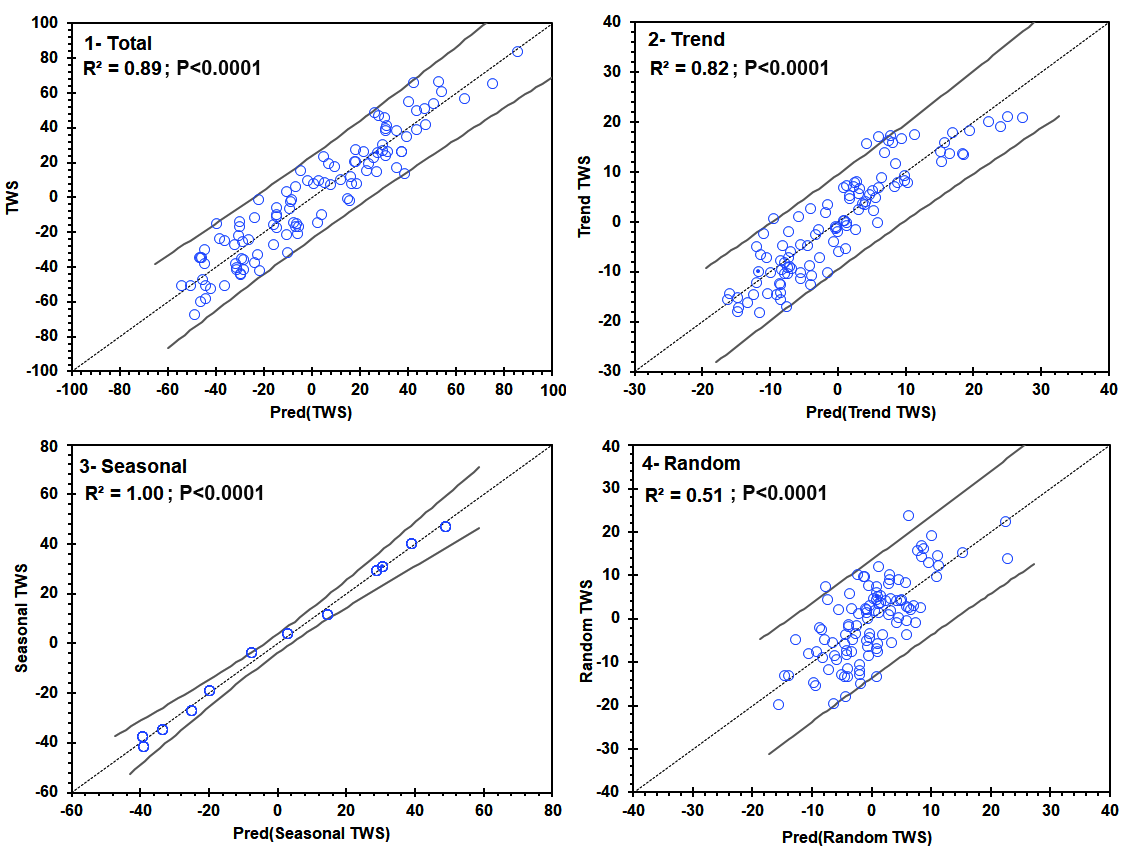


Figure S5. Example of statistical modeling of CSR-M TWS using different time series component from LSM including total, trend, seasonal, and random component.


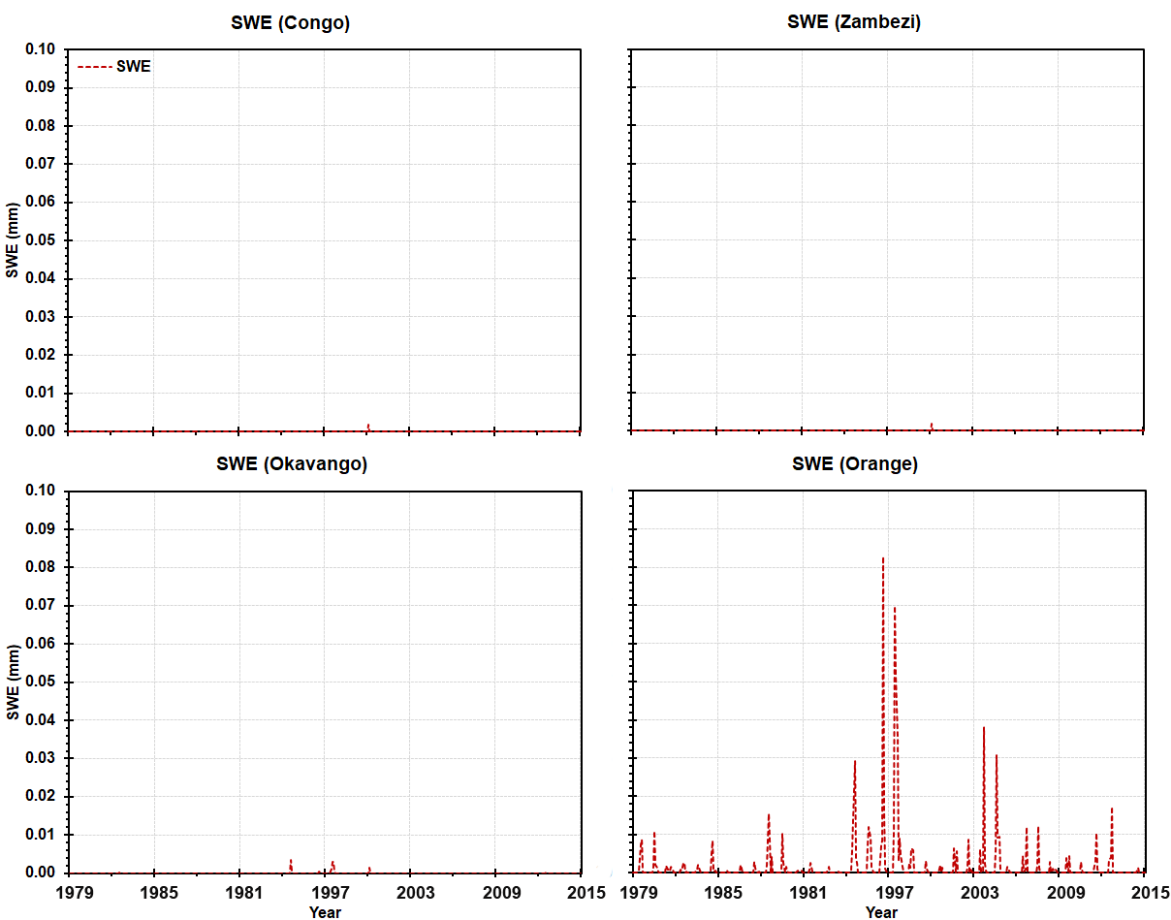


Figure S6. Snow water equivalent (SWE) derived for four studied river basins, most of the values are closer to zero, the maximum SWE estimate is less than 0.1mm for orange river basin.


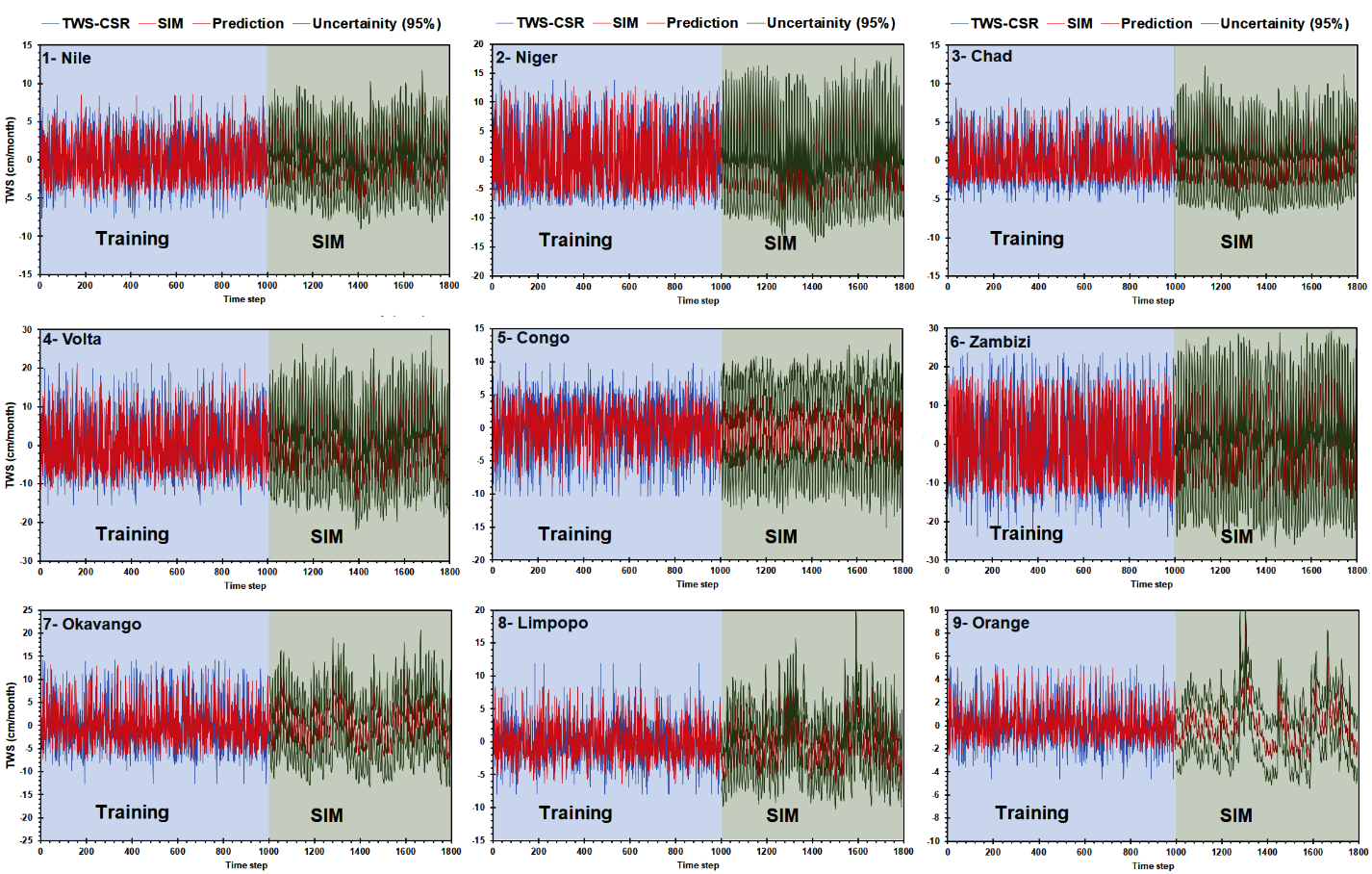


Figure S7. Simulation of TWS variation using a random sample of 1000-point as model training. The random points were generated using the independent (CSR-M) and explanatory variables (LSM+ENSO) between the period from April 2002 – December 2016. First, a linear regression approach was applied to eliminate the linear trend (long-term component), and to generate a detrended time series. The model well-captured the mode of variability in TWS as indicated by the time series generated during the training period, for more details, see Figure S8. The 95 percent confidence interval is indicated around the estimates. Table S9 summaries the goodness-of-fitting coefficient.


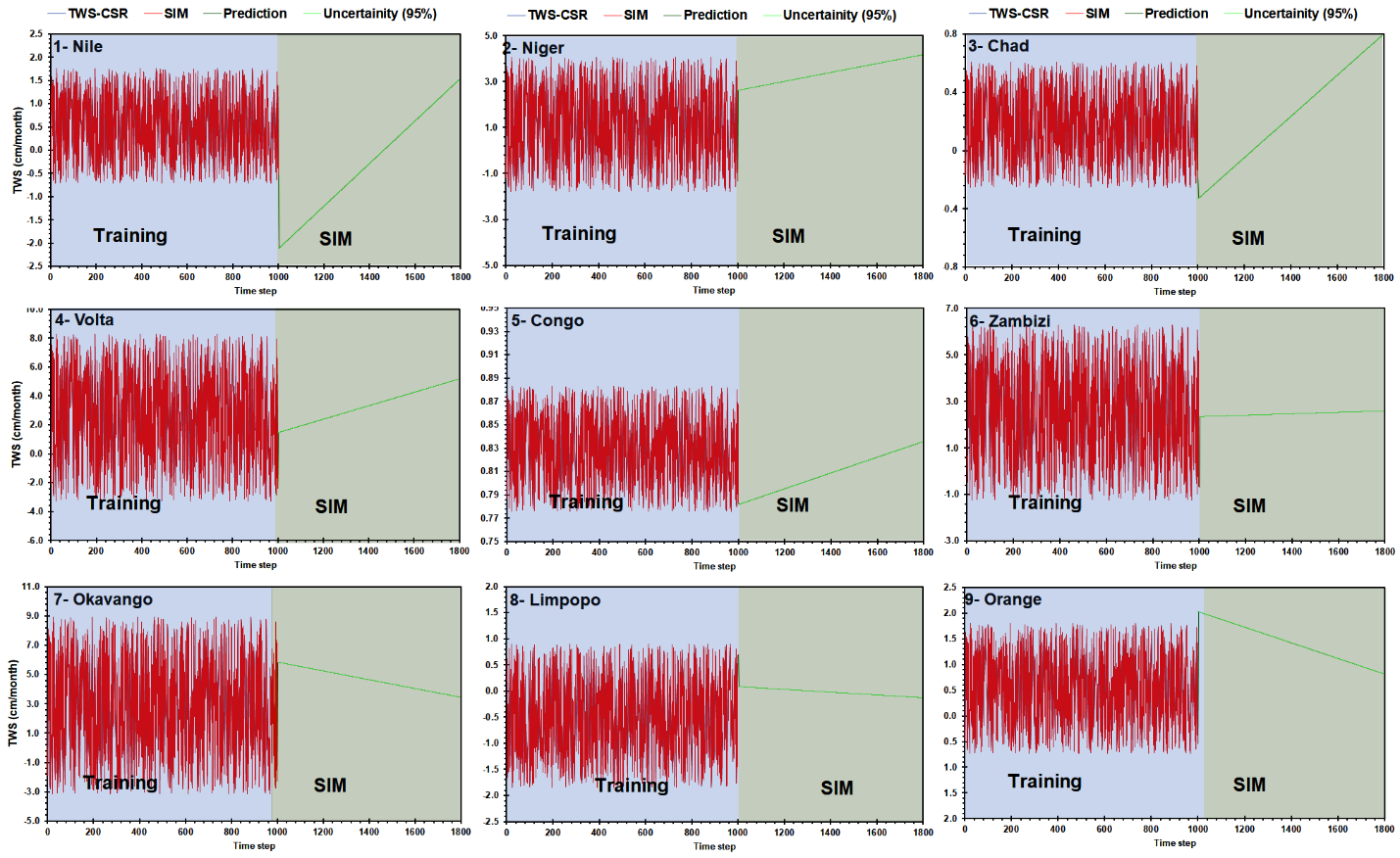


Figure S8. Simulation of the TWS trend component using a random sample of 1000-points as model training. A linear regression applied to estimate the long-term linear trend. The random samples were generated using the trend component from the independent (CSR-M) and explanatory variables (LSM+ENSO) between the period from April 2002 – December 2016.

**Table**

| **Goodness-of-fit** | **Nile** | **Niger** | **Chad** | **Volta** | **Congo** | **Zambezi** | **Okavango** | **Limpopo** | **Orange** |
| --- | --- | --- | --- | --- | --- | --- | --- | --- | --- |
| RMSE | 1.50 | 2.27 | 1.56 | 3.89 | 2.98 | 5.22 | 3.01 | 1.94 | 1.15 |
| WN Variance | 2.26 | 5.17 | 2.43 | 15.13 | 8.88 | 27.29 | 9.06 | 3.77 | 1.32 |
| FPE | 2.27 | 5.18 | 2.44 | 15.16 | 8.90 | 27.34 | 9.08 | 3.78 | 1.32 |
| AICC | 3673 | 4498 | 3744 | 5572 | 5040 | 6162 | 5060 | 4182 | 3134 |
| Standard error | 1.50 | 2.27 | 1.56 | 3.89 | 2.98 | 5.22 | 3.01 | 1.94 | 1.15 |

**Table S1. Summary of model Goodness-of-fit coefficient**

RMSE: Root Mean Square Error; WN Variance: White Noise Variance; FPE: Akaike’s Final Predication Error; AICC: Akaike Information Criterion Corrected.
